# The Switch from Ferric Gluconate to Ferric Carboxymaltose in Hemodialysis Patients Acts on Iron Metabolism, Erythropoietin, and Costs: A Retrospective Analysis

**DOI:** 10.3390/medicina59061071

**Published:** 2023-06-02

**Authors:** Antonio Lacquaniti, Romana Gargano, Susanna Campo, Teresa Casuscelli di Tocco, Silvia Schifilliti, Paolo Monardo

**Affiliations:** 1 Nephrology and Dialysis Unit, Papardo Hospital, 98158 Messina, Italy; ant.lacq@gmail.com (A.L.);; 2 Department of Economics, University of Messina, 98100 Messina, Italy; 3 Faculty of Pharmacy, Clinical Pharmacy Fellowship, University of Messina Annunziata Campus, 98168 Messina, Italy

**Keywords:** erythropoietin resistance index, ferric carboxymaltose, pharmacoeconomics, ESA, transferrin saturation

## Abstract

*Background and Objectives:* Iron deficiency and anemia characterize patients on chronic hemodialysis (HD). Available intravenous iron agents, such as ferric gluconate (FG) and ferric carboxymaltose (FCM), vary in dosing regimens and safety profiles. The aim of the present study was to analyze the modification of the iron status, the correction of anemia, and the economic implications after the shift from FG to FCM therapy in chronic HD patients. We evaluated, during the study, the variations in iron metabolism, assessing ferritin and transferrin saturation, erythropoietin-stimulating agent (ESA) doses and the number of administrations, the effects on anemic status, and consequent costs. *Materials and Methods:* A retrospective study was performed with a follow-up period of 24 months, enrolling forty-two HD patients. The enrolment phase started in January 2015, when patients were treated with iv FG, and continued until December 2015, when FG was discontinued, and, after a wash-out period, the same patients were treated with FCM. *Results:* The iron switch reduced the administered dose of ESA by 1610.500 UI (31% of reduction; *p* < 0.001) during the entire study period and reduced the erythropoietin resistance index (ERI) (10.1 ± 0.4 vs. 14.8 ± 0.5; *p* < 0.0001). The FCM group had the highest percentage of patients who did not require ESA treatment during the study period. The FCM patients were characterized by higher levels of iron (*p* = 0.04), ferritin (*p* < 0.001), and TSAT levels (*p* < 0.001) compared to the FG patients. The annual cost during FG infusion was estimated at EUR 105,390.2, while one year of treatment with FCM had a total cost of EUR 84,180.7 (a difference of EUR 21,209.51 (20%), saving EUR 42.1 per patient/month (*p* < 0.0001). *Conclusions:* FCM was a more effective treatment option than FG, reducing ESA dose requirements, increasing Hb levels, and improving iron status. The reduced ESA doses and the decreased number of patients needing ESA were the main factors for reducing overall costs.

## 1. Introduction

Many patients receiving maintenance hemodialysis (HD) are characterized by a negative iron balance, which is compromised by the upregulation of hepcidin activity occurring in inflammatory states, as revealed in uremic patients [1]. The inadequate iron availability, often observed in these patients, leads to resistance or hypo-responsiveness to erythropoietin-stimulating agent (ESA) [2]. Chronic inflammation and a supply/demand mismatch, secondary to red blood cell overproduction related to ESA, induce an inefficient utilization of iron stores [3,4]. A fine balance between ESA and iron levels, with consequent positive effects, should be the target for the management of anemia, along with the achievement of hemoglobin (Hb) levels, as underlined in the PIVOTAL trial, highlighting cardiovascular benefits with appropriate iron status [5,6]. During the last decades, HD patients received several iv iron preparations, with consequent heterogeneity in administration methods, doses, and duration of treatment, leading to non-homogeneous data. Older iron preparations, such as ferric gluconate (FG), required small single doses administered in short intervals, whereas more stable iron preparations, such as ferric carboxymaltose (FCM), allow for high single doses with personalized intervals of administration [7,8]. Furthermore, in recent years, several studies have focused on the cost-effectiveness and cost–benefit analyses between different iron preparations, both in HD patients and in pre-dialytic chronic kidney disease. In particular, Covic revealed that FCM was well tolerated and effective in the correction of hemoglobin (Hb) levels and iron stores in patients with iron deficiency anemia in HD patients. Similarly, Hofman demonstrated the better management of iron metabolism and anemia with FCM than iron sucrose [9,10]. Our group compared the effects of FCM and ferric gluconate on anemia in HD patients, highlighting a reduction in the erythropoietin (ESA) dose in 4 years of follow-up during FCM treatment, via the better control of anemia mediated by high levels of ferritin and TSAT, and a reduction in the erythropoietin resistance index [11]. 

Similar data were also revealed by other analyses that assessed a reduction in ESA obtained by FCM treatment, with consequent economic advantages [12,13]. However, although FCM appears to be an attractive option in terms of both efficacy and safety, the widespread use of this formulation is not yet supported by a high level of evidence.

The most appropriate IV iron-replacement regimen in adults undergoing dialysis is unknown. The Kidney Diseases Outcomes Quality Initiative (KDOQI) recommended IV iron to support hemoglobin levels between 11 and 12 g/dL, maintaining ferritin between 100 and 800 ng/mL and transferrin saturation (TSAT) between 20% and 50% [14].

However, during the last decade, several studies were published about iron management and anemia in HD patients, proposing different therapeutic strategies and optimizing the dosage of IV iron. In particular, the PIVOTAL (Proactive IV Iron Therapy in Hemodialysis Patients) trial compared a proactive, high-dose IV iron arm (iron sucrose administered at 400 mg/month unless ferritin >700 µg/L and/or TSAT ≥ 40%) and a reactive, low-dose IV iron arm (iron sucrose administered if ferritin <200 µg/L or TSAT < 20%) [15]. 

This study revealed advantages from the proactive approach, assessing not increase risks for stroke and infections in HD patients [16,17]. 

According to these data, which encourage a re-examination of previous recommendations, KDIGO organized the Controversies Conference, which mainly focused on iron-related issues, emerging iron therapies, treatment targets, and patient outcomes [18].

The aim of the present study was to analyze the modification of the iron status, the correction of anemia, and economic implications after the shift from FG to FCM therapy in chronic HD patients. We evaluated, during the study, the variations of iron metabolism, assessing ferritin and transferrin saturation, ESA doses and the number of administrations, the effects on anemic status, and consequent costs.

## 2. Materials and Methods

### 2.1. Study Design

A retrospective study was performed with a follow-up period of 24 months. The enrolment phase started in January 2015, when patients were treated with iv FG (Ferlixit, Aventis Pharma, Milan, Italy). The patients had previously been administered iv FG for ≥6 months. This phase lasted for 12 months, until December 2015, when FG was discontinued. During January 2016, a wash-out period was performed. From February 2016 to February 2017, the same patients were treated with FCM (Ferinject^®^; Vifor (International) Inc., St. Gallen, Switzerland). 

Patients received an undiluted dose of FCM directly into the venous line of the dialyzer ~30–60 min into the dialysis session, and we evaluated Hb, transferrin saturation (TSAT), and ferritin levels, following the guidelines of the KDIGO [14]. iv iron therapy was initiated when there was an absolute iron deficiency, defined by TSAT < 20% or ferritin < 200 ng/mL.

For maintenance therapy, at monthly hematic control, if ferritin was 200–500 μg/L and/or TSAT was 20–30%, the FCM dose was 100 mg every two weeks. If ferritin was 500–800 μg/L and/or TSAT was 30–50%, the FCM dose was 100 mg monthly. If ferritin was > 800 μg/L and/or TSAT was >50%, no IV iron was administered.

The same modification could be applied for FG, but all FG patients always required 125 mg per week to obtain an adequate iron metabolism. Similarly to FCM, FG was directly infused into the venous line of the dialyzer.

All patients received epoetin zeta (Epo-z) (Retacrit^®^, Pfizer Italy s.r.l. Latina, Italy), a biosimilar recombinant human erythropoietin preparation of epoetin alfa, for the entire observation period, if necessary.

For cost estimation, data were obtained from the hospital administrative office and from the online website of the Italian Ministry of Economy and Finance: https://contoannuale.rgs.mef.gov.it/ (accessed on 9 January 2023).

### 2.2. Patients and Baseline Data

A total of 55 patients, receiving maintenance HD as outpatients at the Papardo Hospital, Nephrology and Dialysis Unit, Messina, Italy, were enrolled. All patients received HD three times a week for 4 h, and the dialysis method was maintained during the entire observation period. Patients were at least 18 years old, were on uninterrupted HD treatment for a period of at least 6 months before the shift from FG to FCM, and continued the treatment for at least 12 months.

Patients with any malignancy diagnosed in the five years before the investigational period were excluded. Moreover, other exclusion criteria included hospitalization during 6 months before the enrolment, hospitalization during the study period, history of gastrointestinal bleeding, history of hemotransfusion, hemoglobin concentrations more than 12.0 g/dL, known hypersensitivity to any component of FCM, anemia other than that due to iron and EPO deficiency, evidence of active infection (including hepatitis B, hepatitis C, or HIV), concomitant severe liver or cardiovascular diseases, and iron storage disorders. 

**Figure 1:**
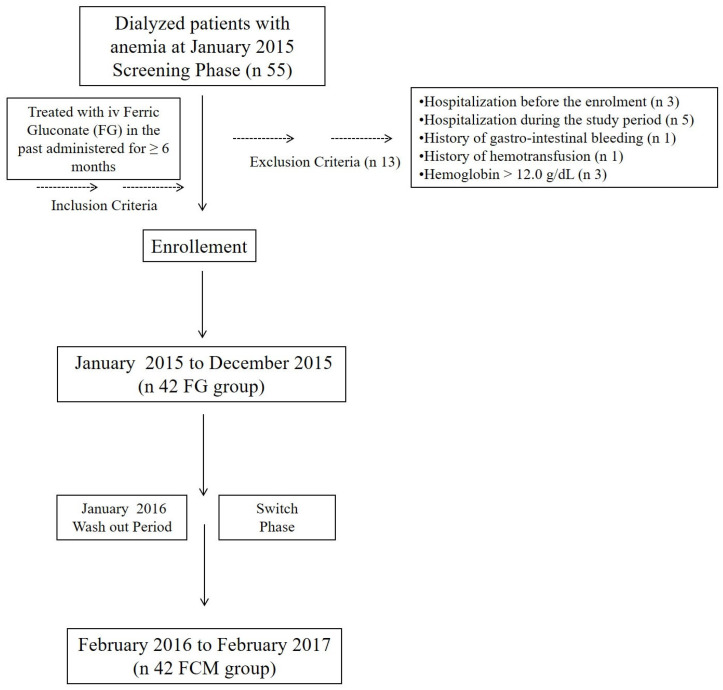
Study design: inclusion and exclusion criteria; enrollment; and switch phase from ferric gluconate to ferric carboxymaltose.

According to the above-mentioned criteria, 42 patients were included in the study (Figure 1).

This study protocol was reviewed and approved by the Ethics committee of the University Hospital of Messina, approval number 29–20. All patients provided informed consent permitting data sampling and analysis at the time of initiation of the dialysis therapy. 

### 2.3. Blood Collection and Biochemical Data

Data were measured once per month, and blood sampling was performed at the beginning of each week when initiating HD. Transferrin saturation (TSAT) was calculated according to the following formula: (serum iron/serum transferrin) × 70.9.

The erythropoietin resistance index (ERI) was defined as the weekly weight-adjusted EPO dose (U/kg/week) divided by the hemoglobin level (g/dL) and calculated monthly to investigate resistance to EPO treatment [19].

### 2.4. Statistical Analysis

Numerical variables are summarized using means and standard error, and 95% confidence intervals and categorical variables are summarized using numbers and percentages. Comparison between groups was performed using non-parametric combination test (NPC test 2.0—Statistical software for multivariate permutation test, Methodologica S.r.l., Treviso, Italy) [20]. 

The NPC methodology works via a decomposition of the k-dimensional multivariate hypothesis testing problem (where k is the number of outcomes) in which the solution to the problem is obtained by employing a two-phase algorithm: In the first phase, an appropriate set of tests of univariate permutation is specified; these are called partial tests. Each partial test is aimed at determining the marginal contribution of each result in the comparison between the different treatment groups (FG and FCM). The second phase consists of the non-parametric combination of the partial tests in a single combined test called second order, which verifies whether there are global differences between the multivariate distributions of the group results. In the event of the presence of a stratification variable defined on s levels (in this case, time = 12), the algorithm presents a further phase since we are in the presence of two levels of combination: the first relates to the combination of the partial tests in s-combined second-order tests, each corresponding to a given stratum, while the second level is defined by a further combination of the stratum tests into a single combined third-order global test. A *p*-value < 0.05 was considered significant.

## 3. Results

### 3.1. Patients Baseline Characteristics

Overall, 42 patients, 67% males and 33% females, with a mean age at enrolment of 62.4 ± 19.6 years, were evaluated. The median value for dialysis vintage was 81 months (range: 48–167). The primary disease was diabetic nephropathy in 16 patients (38%), nephrosclerosis in 12 patients (29%), chronic glomerulonephritis in 8 patients (19%), and other causes in the remaining 6 patients (14%). Each dialysis session lasted 4 h with a mean weekly spKt/V of 1.43 ± 0.4. Moreover, arteriovenous fistula represented the vascular access for 78% of patients, whereas the remaining 22% were dialyzed via a central venous catheter. All patients underwent hemodiafiltration in post-dilution mode with a mean blood flow rate of 300 ± 20 mL/min. The mean Hb, serum iron, ferritin, and TSAT were 11.3 ± 1.3 g/dL, 94.5 ± 41.7 μg/dL, 566.2 (345–758) ng/mL, and 21.8 ± 9.5%, respectively. No adverse systemic side effects, such as hemodynamic alterations, cutaneous rush, dizziness, or itch, were recorded after iron administration during the entire study period. Moreover, we did not observe an excessive increase in hemoglobin, hematocrit, or ferritin and, thus, did not alter the function of the vascular access.

### 3.2. Epo and ERI Modifications

When patients received FCM, they were characterized by higher levels of serum iron (+6.8; 95% CI: −1.8 +15.3; *p* = 0.04) and ferritin (+153.4; 95% CI: +81.5, +222.7; *p* < 0.001) compared to during FG, while transferrin values were reduced (−12.8; *p* = 0.001). Furthermore, FCM increased TSAT levels by 10% (*p* < 0.001) compared to FG. 

This trend was closely related to ERI reduction, for which levels were more diminished with FCM than with FG (10.1 ± 0.4 vs. 14.8 ± 0.5; *p* < 0.0001), especially after six months of FCM therapy. Table 1.

**Figure 2:**
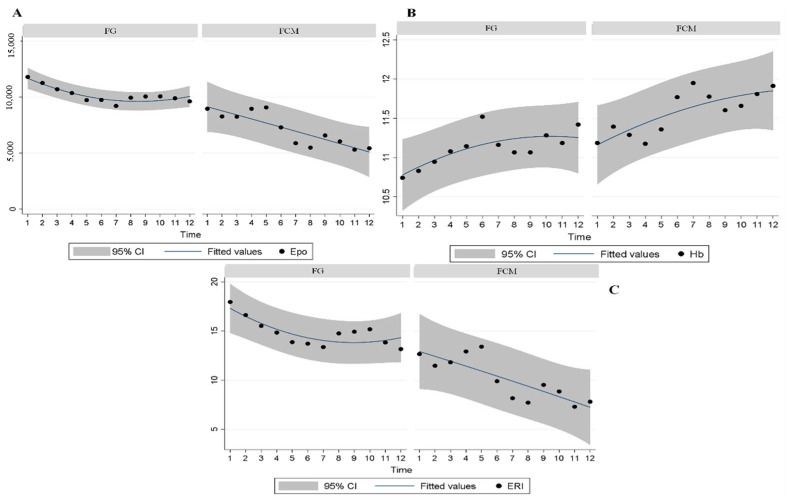
Trends in Epo, HB, and ERI mean fitted values and 95% confidence intervals for each treatment. (**A**) Trends in Epo with FG and FCM Treatment; (**B**) Trends in HB with FG and FCM Treatment; (**C**) Trends in ERI with FG and FCM Treatment.

During the entire study period, Hb levels were maintained within recommended ranges, with best values recorded after six months of FCM treatment (11.2 ± 0.05 vs. 11.5 ± 0.05; *p* < 0.001), as the expression of improved iron status, notwithstanding reduced overall Epo-z doses (10,207.1 ± 312.8 UI vs. 7150.7 ± 278.1 UI). Figure 2, Table 2. 

**Figure 3:**
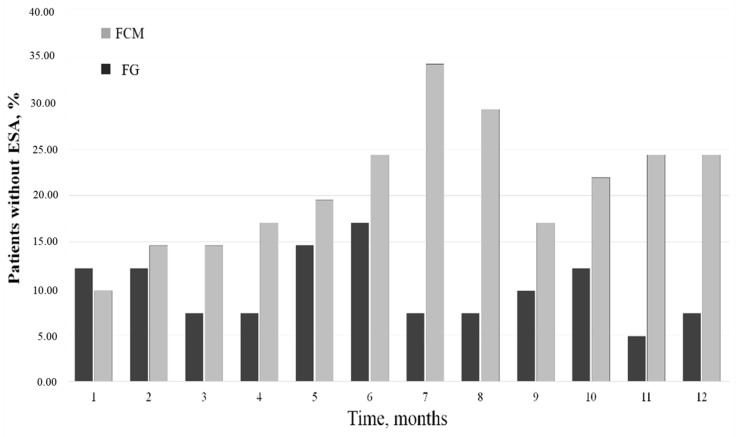
Percentage of patients without Epo therapy.

Moreover, as underlined by Figure 3, FCM showed the highest percentage of patients who did not require Epo-z treatment during the study period. In particular, at the seventh month after the switch, 33% of patients receiving FCM did not receive erythropoietin, whereas the same patients, during the FG therapy, were treated with EPO-z (only 5% of these patients were not). This trend was shown in the FCM cohort with different percentages during the remaining months of observation. In the group of patients not treated with EPO-z, we assessed higher ferritin levels than in patients receiving erythropoietin (688.5 (567.8–812.4) vs. 323.4 (221.5–589.1) ng/mL; *p* < 0.01), as well as higher TSAT values (31.3 ± 5.2% vs. 22.8 ± 6.1%; *p*: 0.01).

In particular, at least six months of FCM therapy induced a significant decrease in the Epo-z dose, as reported in Table 3. 

### 3.3. FG vs. FCM: Dosages and Costs

The entire cohort of patients, in the pre-switch phase, received 3976 FG vials, whereas 1345 FCM vials were administered in the post-switch period, with increased costs related to iron formulation in the latter period (EUR 5213.2 vs. EUR 1528; *p* < 0.001). 

However, when receiving FG, the cohort received 248.500 mg of elemental iron, considering that a 5 mL vial contains 62.5 mg of elemental iron. During the FCM therapy, the cohort received a smaller dosage of elemental iron (134.500 mg, *p* < 0.01), obtaining an improved iron metabolism. In fact, the iron switch reduced the Epo-z dose by 1610.500 UI (31% of reduction; 5215.500 UI vs. 3605.000 UI Epo-z; *p* < 0.001) during the entire study period.

During the FG treatment, the average monthly cost per patient was EUR 145.50, whereas after the switch, the average cost due to Epo-z therapy was EUR 100.7, with savings of about 30.8% (*p* < 0.001).

**Figure 4:**
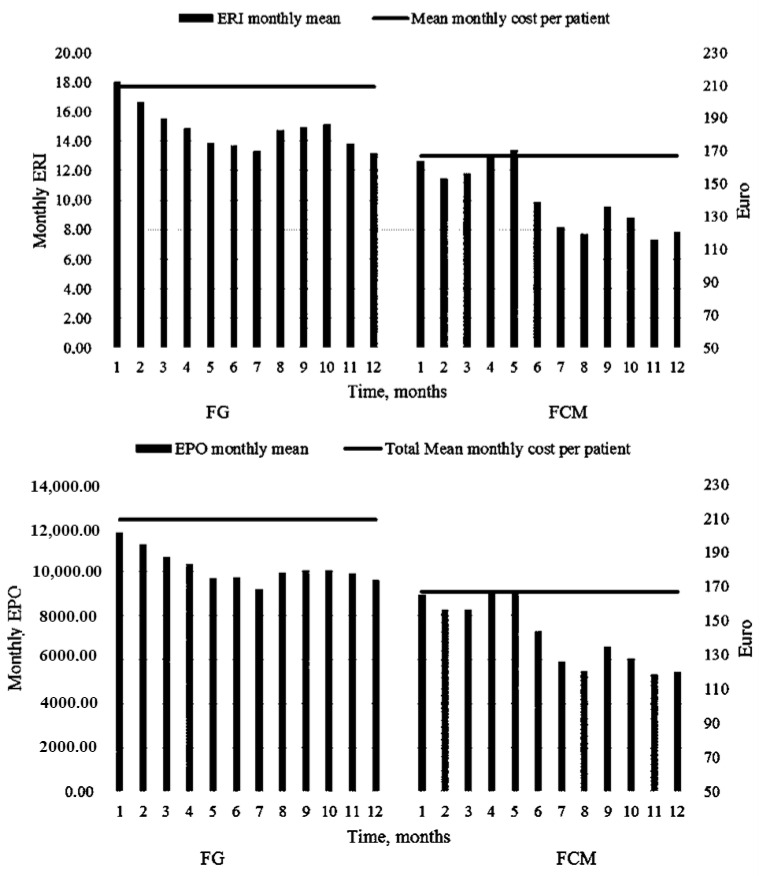
Monthly ERI value and Epo cost pre- and post-switch.

Figure 4 displays the evaluated mean cost for Epo-z during the study period. 

The annual cost during FG infusion, including Epo-related fees, was estimated at EUR 105,390.2 (on average, EUR 209.1 per patient/month), while one year of treatment with FCM had a total cost of EUR 84,180.7 (on average, EUR 167.03 per patient/month). The difference between the two treatments was EUR 21,209.51 (20%), saving EUR 42.1 per patient/month (*p* < 0.0001). Table 4. 

Excluding the fixed cost of personnel, 12 months of the FG treatment cost EUR 76,956.87 (EUR 152.69 per patient/month), while the FCM treatment cost EUR 56,662.7 (EUR 112.43 per patient/month), recording savings of 26.3%, corresponding to EUR 20,294.15 (EUR 40.27 per patient/month).

## 4. Discussion

This study evaluated the cost-effectiveness of iron treatments in anemic HD patients, demonstrating that FCM was the more effective treatment option compared to FG, reducing ESA dose requirements, increasing Hb levels, and improving iron status. In fact, when receiving FCM, the cohort was characterized by a reduced percentage of patients with a TSAT of <20%. The increased ferritin and TSAT values, observed during FCM therapy, are related to an improved bioavailability of elemental iron and a gradual and more stable release of iron into the blood, which helped to avoid transferrin saturation. Moreover, reduced transferrin values were observed after FCM therapy. Transferrin has a high affinity to ferric iron, and, therefore, low transferrin in plasma indicates iron overload, which means the binding site of transferrin is highly saturated with iron. This evidence reflects the positive effects of FCM on iron metabolism.

The main factors for reducing overall costs were the reduced ESA doses and the decreased number of patients needing ESA at the end of the follow-up period.

In particular, the economic starting point was unfavorable for the FCM choice due to its high costs, notwithstanding the reduced number of vials used compared to FG. However, after six months of therapy, this gap was neutralized and overturned due to a better and personalized response in terms of TSAT and Hb levels. These results strengthen the evidence emerging from the literature about the effects of FCM in uremic patients and the related implications from an economic point of view [9,12,21,22]. 

However, different points distinguish our data. Severe Hb levels and severe iron deficiency did not characterize our cohort. The very strict inclusion criteria, excluding hemotransfused patients, were intended to evaluate the effects of the two iron formulations, bypassing enormous bias due to blood transfusion or very critical anemia, which, along with ESA or iron deficiency, could also suggest multifactorial etiologies. Patients on target for Hb levels and iron status during FG therapy were analyzed. No patients during the study period received red cell transfusions, often contributing to iron overload and immunological issues in HD patients waiting for renal transplantation. The United States Renal Data System data have reported that blood transfusion is still widely used for anemia management in HD patients, despite the widespread use of ESA [23]. 

These data likely reflect a lack of attention toward the iron metabolism and inflammatory processes in HD patients, which often induce ESA resistance. 

FCM allows the personalization of prescriptions, achieving and maintaining iron and Hb targets with reduced costs. The maintenance of increased ferritin values, which are higher during FCM treatment than during FG treatment, highlights different pharmacokinetic actions mediated by the gradual and low release of FCM iron into the bloodstream, preventing hepcidin-induced iron sequestration and oxidative stress [24].

Other studies have shown more reductions in the amount of ESA by iv iron supplementation required to increase ferritin to higher than 300 ng/mL and TSAT to 30–50% [25,26]. 

As for iron overload concerns, high ferritin levels were not associated with adverse events in the non-HD nephropathic population treated by FCM [27]. 

In the Dialysis Patients Response on IV Iron with Elevated Ferritin (DRIVE) study, an intensive iv iron administration protocol (125 mg ferric gluconate for eight HD sessions) can significantly reduce ESA dosing requirements in patients who have a ferritin level > 500 ng/mL and a TSAT < 25% when also receiving adequate epoetin [28].

The Renal Association increased the ferritin ceiling to 800 ng/mL during iron supplementation therapy, proposing this cut-off level as the threshold for withholding IV iron supplementation in HD patients [29,30]. However, these safety findings, albeit reassuring, should not be generalized, considering the close relationship between high ferritin levels and inflammation in HD patients [31,32]. 

Iron deficiency in cardiac heart failure has only recently drawn clinical attention, predicting poor outcomes that are independent of anemia. Several studies have revealed significant differences in terms of cardiovascular events between HD patients treated with FCM and HD patients treated with other iron formulations, with potential dose-sparing effects of FCM on EPO [6,33].

Our observational period was 12 months for each group, with ESA reduction obtained only six months after the shift. This datum could be associated with a close link between improved iron status and ERI levels, for which values were more reduced during FCM treatment than during FG treatment.

Our cohort reached high levels of ferritin and TSAT, but one year of observation is a short period of time, which does not allow us to reach conclusions about cardiovascular events. However, during FCM treatment, the group did not reach values of ferritin < 200 ng/mL and TSAT < 20%, which are associated with increased cardiovascular mortality [34].

Our study revealed no adverse systemic side effects, such as hemodynamic alterations, cutaneous rush, and itch, after iron administration during the entire study period and did not have an excessive increase in hemoglobin, hematocrit, or ferritin.

The same biosimilar Epo-z was administered, obtaining a homogeneous group and avoiding different responses that could be related to different ESA formulations. If all ESA formulations were bioequivalent, without clinically relevant differences between originators and biosimilars, the biosimilar of epoetin alpha, compared to the corresponding originator, had a reduced activity of 35% in clinical practice [35].

Moreover, the benefits with FCM, obtaining the highest percentage of patients who did not require Epo-z treatment, have important clinical implications, beyond the economic impact. 

In particular, at the seventh month after the switch, 33% of patients did not receive erythropoietin therapy due to improved iron metabolism and bioavailability, and this trend was confirmed during the following months of observation. 

Large randomized, controlled clinical trials in nephropathic patients have raised safety concerns associated with high-dose ESA therapy and high Hb targets, citing death and hospitalization for chronic heart failure and fatal or nonfatal stroke [36,37].

Moreover, the risks of vascular access thrombosis and cancer have been associated with high ESA doses [38,39]. 

The present study has some limitations that should be mentioned. First, it was a single-center and retrospective study, and the cohort of patients was relatively small. However, this study selected patients in a steady state on a stable iron dosing regimen, to reduce potential bias, such as red blood transfusions or different degrees of anemia, obtaining homogeneous groups. A long period of follow-up has been also conducted, but a prospective study evaluating the effects of FCM on Hb levels and iron metabolism is required, and confirmation in wider cohorts is indispensable to attribute general validity to our reports. Despite these limitations, this study provides an estimate of the potential savings from the use of FCM for the treatment of anemic HD patients.

## 5. Conclusions

The ferric gluconate therapy maintained hemoglobin levels and iron metabolism markers on target but required high doses of erythropoietin-stimulating agent. Ferric carboxymaltose induced a boost of ferritin and transferrin saturation values, reducing both the total amount of erythropoietin administered and the number of patients dependent on it. The economic effect was significant with a not negligible reduction in costs. However, confirmations in wider cohorts are indispensable due to the limitation of this retrospective study, which was based on a relatively small cohort of patients. A new economic evaluation will be required when new drugs will be available for nephrologists for the management of anemia in hemodialyzed patients, such as Hypoxia-inducible factor-1. Uremic patients require careful monitoring for inflammation status, which is often responsible for erythropoietin resistance and inadequate iron availability.

## Figures and Tables

**Table 1:** Erythropoietin resistance index (ERI) for each month before and after switching treatments.

F/U	FG Treatment		FCM Treatment		
Time	Mean ± SD	95% CI	Mean ± SD	95% CI	*p*-Value
1	17.9 ± 2.0	14.0–21.9	12.6 ± 1.5	9.6–15.7	0.036
2	16.6 ± 1.8	12.9–20.3	11.4 ± 1.5	8.4–14.5	0.037
3	15.5 ± 1.3	12.8–18.2	11.8 ± 1.7	8.4–15.1	0.094
4	14.8 ± 1.5	11.8–17.8	12.9 ± 1.5	9.9–15.9	0.380
5	13.8 ± 1.8	10.1–17.5	13.4 ± 1.9	9.5–17.3	0.865
6	13.7 ± 2.0	9.7–17.7	9.9 ± 1.6	6.7–13.0	0.154
7	13.3 ± 1.5	10.2–16.5	8.1 ± 1.2	5.7–10.6	0.011
8	14.7 ± 1.8	11.1–18.3	7.7 ± 1.2	5.2–10.2	0.001
9	14.9 ± 1.8	11.2–18.6	9.5 ± 1.4	6.7–12.3	0.022
10	15.1 ± 2.0	11.1–19.2	8.8 ± 1.3	6.2–11.4	0.012
11	13.8 ± 1.2	11.3–16.3	7.3 ± 1.0	5.3–9.3	<0.001
12	13.1 ± 1.5	10.1–16.2	7.8 ± 1.6	4.6–11.0	0.014

Abbreviations: F/U: follow-up time in months; FG: ferric gluconate; FCM: ferric carboxymaltose; SD: standard deviation; CI: confidence interval.

**Table 2:** Hb level for each month before and after switching treatments.

F/U	FG Treatment		FCM Treatment		
Time	Mean ± SD	95% CI	Mean ± SD	95% CI	*p*-Value
1	10.7 ± 0.2	10.2–11.2	11.1 ± 0.2	10.7–11.6	0.185
2	10.8 ± 0.2	10.3–11.2	11.3 ± 0.2	11.0–11.7	0.068
3	10.9 ± 0.2	10.5–11.3	11.2 ± 0.2	10.8–11.7	0.251
4	11.0 ± 0.1	10.7–11.4	11.1 ± 0.1	10.8–11.5	0.688
5	11.1 ± 0.1	10.8–11.4	11.3 ± 0.2	10.8–11.8	0.454
6	11.5 ± 0.1	11.1–11.8	11.7 ± 0.1	11.4–12.0	0.290
7	11.1 ± 0.1	10.8–11.5	11.9 ± 0.1	11.6–12.2	0.001
8	11.0 ± 0.1	10.7–11.3	11.7 ± 0.1	11.4–12.0	0.002
9	11.0 ± 0.1	10.6–11.4	11.6 ± 0.1	11.2–11.9	0.033
10	11.2 ± 0.1	10.9–11.6	11.6 ± 0.1	11.3–11.9	0.138
11	11.1 ± 0.1	10.8–11.5	11.8 ± 0.1	11.4–12.2	0.013
12	11.4 ± 0.1	11.1–11.7	11.9 ± 0.6	11.5–12.2	0.049

Abbreviations: F/U: follow-up time in months; FG: ferric gluconate; FCM: ferric carboxymaltose; SD: standard deviation; CI: confidence interval.

**Table 3:** Epo level for each month of study before and after switching treatments.

F/U	FG Treatment		FCM Treatment		
Time	Mean ± SD	95% CI	Mean ± SD	95% CI	*p*-Value
1	11,804.8 ± 1240.1	9371.1–14,238.6	8963.4 ± 1044.2	6914.2–11,012.5	0.090
2	11,268.2 ± 1255.2	8805.0–13,731.5	8280.4 ± 1068.1	6184.4–10,376.5	0.076
3	10,707.3 ± 1020.0	8705.5–12,709.0	8256.1 ± 1061.6	6172.6–10,339.5	0.107
4	10,365.8 ± 1028.1	8348.3–12,383.4	8963.4 ± 1006.7	6987.8–10,939.0	0.348
5	9731.7 ± 1164.5	7446.4–12,016.9	9085.3 ± 1148.4	6831.7–11,339.0	0.701
6	9756.1 ± 1288.3	7227.9–12,284.3	7304.8 ± 1061.0	5222.6–9387.1	0.164
7	9219.5 ± 965.1	7325.6–11,113.4	5890.2 ± 865.9	4190.9–7589.5	0.011
8	9951.2 ± 1044.2	7901.9–12,000.4	5500.0 ± 802.4	3925.3–7074.6	<0.001
9	10,060.9 ± 1047.0	8006.1–12,115.7	6585.3 ± 837.3	4942.1–8228.6	0.011
10	10,073.1 ± 1102.3	7909.9–12,236.3	6048.7 ± 830.0	4419.9–7677.6	0.004
11	9900.0 ± 819.4	8291.8–11,508.1	5317.0 ± 667.5	4007.0–6627.1	<0.001
12	9625.0 ± 998.5	7665.5–11,584.4	5439.0 ± 842.5	3785.70–7092.3	0.001

Abbreviations: F/U: follow-up time in months; FG: ferric gluconate; FCM: ferric carboxymaltose; SD: standard deviation; CI: confidence interval.

**Table 4:** Healthcare resources total costs for the FG and FCM periods.

Resource	Unit cost	
FCM *	EUR 3.876/mL (100 mg)	
FG *	EUR 0.38431/fL (62.5 mg)	
Syringe *	EUR 0.034	
Infusion set *	EUR 0.093	
Physiological solution 100 mL *	EUR 0.396	
Epo-z 2000 UI *	EUR 28.09	
Epo-z 3000 UI *	EUR 42.15	
Epo-z 4000 UI *	EUR 56.23	
Epo-z 5000 UI *	EUR 70.28	
Epo-z 6000 UI *	EUR 84.30	
Epo-z 8000 UI *	EUR 112.34	
Epo-z 10,000 UI *	EUR 140.58	
Personal time—physician (1 h) ^#^	EUR 41.50	
Personal time—nurse (1 h) ^#^	EUR 15.70	
Cost item in EUR	FG	FCM
Iron treatment	1528	5213.2
Epo-z	73,349.4	50,746.1
Infusion material	2079.4	703.4
Personnel	28,433.3	27,517.9
Total	105,390.2	84,180.7

* Data obtained from hospital administrative office; # data obtained from https://www.contoannuale.mef.gov.it (accessed on 9 January 2023). Abbreviations: FG: ferric gluconate; FCM: ferric carboxymaltose; Epo-z: erythropoietin zeta.

## Data Availability

The dataset generated and analyzed during the current study is available from the corresponding author on reasonable request.
